# Supplementary material for: Local hypergraph clustering using capacity releasing diffusion
Source: PLoS One. 2020 Dec 23;15(12):e0243485. doi: 10.1371/journal.pone.0243485 (PMC7757905; doi:10.1371/journal.pone.0243485)
Supplement: S1 Table — Each column represents a community in a SNAP dataset, for example DBLP-104 means the community number 104 in com-DBLP dataset. We report the median of F1 and the violin plots show the distribution while varying the seed node. (PDF) [file pone.0243485.s001.pdf]

**S1 Table.** Comparison between CRD, CRD-M, HG-CRD, APPR and MAPPR using SNAP datasets. Each column represents a community in a SNAP dataset, for example DBLP-104 means the community number 104 in com-DBLP dataset. We report the median of F1 and the violin plots show the distribution while varying the seed node.

| Alg | DBLP-104<br>F1 | DBLP-487<br>F1 | DBLP-595<br>F1 | Amazon-626<br>F1 | Amazon-649<br>F1 |
|---|---|---|---|---|---|
| HGCRD | **0.93** | 0.32 | **0.75** | 0.99 | **0.99** |
| CRDM | 0.68 | 0.29 | 0.09 | **1.0** | **0.99** |
| CRD | 0.48 | 0.32 | 0.12 | 0.99 | **0.99** |
| APPR | 0.65 | **0.33** | 0.16 | 0.99 | **0.99** |
| MAPPR | 0.75 | 0.29 | 0.13 | **1.0** | **0.99** |
