# Supplementary material for: Local hypergraph clustering using capacity releasing diffusion
Source: PLoS One. 2020 Dec 23;15(12):e0243485. doi: 10.1371/journal.pone.0243485 (PMC7757905; doi:10.1371/journal.pone.0243485)
Supplement: S2 Table — Each column represents a community in a SNAP dataset, for example DBLP-104 means the community number 104 in com-DBLP dataset. We report the median of motif conductance and the violin plots show the distribution while varying the seed node. (PDF) [file pone.0243485.s002.pdf]

S2 Table. Comparison between CRD, CRD-M, HG-CRD, APPR and MAPPR using SNAP datasets. Each column represents a community in a SNAP dataset, for example DBLP-104 means the community number 104 in com-DBLP dataset. We report the median of motif conductance and the violin plots show the distribution while varying the seed node.

Alg	DBLP-104<br>Motif Cond.	DBLP-487<br>Motif Cond.	DBLP-595<br>Motif Cond.	Amazon-626<br>Motif Cond.	Amazon-649<br>Motif Cond.
HGCRD	**0.02**	0.09	0.15	**0.01**	**0.0**
CRDM	**0.02**	0.06	0.08	**0.01**	**0.0**
CRD	0.06	0.06	0.1	**0.01**	**0.0**
APPR	0.05	0.06	0.08	**0.01**	**0.0**
MAPPR	**0.02**	**0.03**	**0.07**	**0.01**	**0.0**
