# Supplementary material for: Local hypergraph clustering using capacity releasing diffusion
Source: PLoS One. 2020 Dec 23;15(12):e0243485. doi: 10.1371/journal.pone.0243485 (PMC7757905; doi:10.1371/journal.pone.0243485)
Supplement: S1 File — (PDF) [file pone.0243485.s003.pdf]

S1 File

Lemma 6.1. *Let* $volM(S_i) = \sum_{u \in V} \mathbf{d}_M(u)$ *be the sum of the motif-based degrees then* $volM(S_i) = (k-1)vol_M(S_i)$.

Proof. This is just algebra:
$\text{volM}(S_i) = \sum_{u \in S_i} \mathbf{d}_M(u) = (k-1) \sum_{u \in S_i} \sum_{e \in \mathcal{E}} \mathbb{1}_{u \in e} = (k-1)\text{vol}_M(S_i)$. □

Theorem 3 (**Theorem 1 [30] Generalization**)**.** *Given* G, $\mathbf{m}_M$, $\phi \in (0,1]$ *such that:* $\mathbf{e}^T \mathbf{m}_M \leq volM(G)$ *and* $\mathbf{m}_M(v) \leq 2\mathbf{d}_M(v) \;\; \forall v \in V$ *at the start, HG-CRD inner terminates with one of the following:*

- ***Case 1:*** *HG-CRD-inner finishes the full HG-CRD step. A full HG-CRD step means that* $\mathbf{m}_M(v) \leq \mathbf{d}_M(v) \; \forall v \in V$.
- ***Case 2:*** *There are nodes with excess and we can find cut* A *of motif-based conductance of* $O(k\phi)$*. Moreover,*
  $2\mathbf{d}_M(v) \geq \mathbf{m}_M(v) \geq \mathbf{d}_M(v) \; \forall v \in A$ *and* $\mathbf{m}_M(v) \leq \mathbf{d}_M(v) \; \forall v \in \bar{A}$.

Proof. We extend the original CRD proof for higher order patterns. Let us classify the nodes into three categories based on their level values:

- **Category 0:** if $\mathbf{l}(v) = h,$ then $2\mathbf{d}_M(v) \geq \mathbf{m}_M(v) \geq \mathbf{d}_M(v) + (k-1)$. Node v kept increasing its level because it had excess of flow until it reached the maximum level.
- **Category 1:** if $h > \mathbf{l}(v) \geq 1,$ then $(k-1) + \mathbf{d}_M(v) > \mathbf{m}_M(v) \geq \mathbf{d}_M(v)$. Node v does not have excess at the end, otherwise, its level would have increased to h.
- **Category 2:** if $\mathbf{l}(v) = 0,$ then $\mathbf{m}_M(v) < \mathbf{d}_M(v) + (k-1)$. Node v never had excess of flow to push.

Proof of case 1: Let $B_i = \{v|l(v) = i\}$.

- If B_h is empty, then the nodes were able to diffuse all of their excess. Then a full HCRD step is done and $\mathbf{m}_M(v) \leq \mathbf{d}_M(v) \ \forall \ v \in V$.
- If B_0 is empty, then level falls in case 0 or case 1 in the previous level categories and as $\mathbf{e}^T\mathbf{m}_M \leq \text{volM}(G)$, then it must be that $\mathbf{m}_M(v) = \mathbf{d}_M(v)$ because $\mathbf{e}^T\mathbf{m}_M = \sum_{u \in V} \mathbf{m}_M(u) = \sum_{u \in V} \mathbf{d}_M(u) = \text{volM}(G)$ and hence full HCRD inner is done and $\mathbf{m}_M(v) \leq \mathbf{d}_M(v) \ \forall \ v \in V$.

Proof of case 2: In this case, B_0 and B_h are not empty and let S_i be the set of nodes with level at least i. The claim will be that one of the S cuts must have conductance $\text{O}(k\phi)$. Let us start by dividing the hyperedges between S_i and $\bar{S}_i$ into two groups:

- **Group 1**: Hyperedges with at least one endpoint in B_j and at least another endpoint in B_j or B_{j-1}, where $j \geq i$.
- **Group 2**: Hyperedges across more than one level (The difference in level values between the node with the highest level to all other nodes in the hyperedge is at least two).

Additionally, let $z_1(i,j) = (k-1) \times |\text{hyperedges in group 1}|$, $z_2(i) = (k-1) \times |\text{hyperedges in group 2}|$, $\phi_1(i,j) = \frac{z_1(i,j)}{\text{volM}(S_i)}$ and $\phi_2(i) = \frac{z_2(i)}{\text{volM}(S_i)}$.
First, we will show that there exists i^* between h and $\frac{h}{2}$ such that: $\phi_1(i^*, j) \leq \frac{\phi}{h}$. This will be a proof by contradiction: Let $\phi_1(i,j) > \frac{\phi}{h} \ \forall i = h, ..., \frac{h}{2}$ and $j \geq i$, then:

$$\begin{aligned} \text{volM}(B_j) &\geq z_1(i,j) \\ \text{volM}(S_{j-1}) &\geq \text{volM}(S_j) + \phi_1(i,j)\text{volM}(S_i) \\ \text{volM}(S_{j-1}) &> (1 + \frac{\phi}{h})\text{volM}(S_j) \qquad \text{using volM}(S_i) \geq \text{volM}(S_j). \end{aligned}$$

As $h = \frac{3 \log \, (\mathbf{e}^T\mathbf{m}_M)}{\phi} \leq \frac{3 \log \, (\text{volM}(G))}{\phi}$, we get:

$$\text{volM}(S_{h/2}) > (1 + \frac{\phi}{h})^{h/2}\text{volM}(S_h) > \Omega((\mathbf{e}^T\mathbf{m}_M)^{3/2}).$$

However, we know that $\text{volM}(S_{h/2}) \leq \mathbf{e}^T\mathbf{m}_M$, which is a contradiction. Therefore, there exists i^* between h and $\frac{h}{2}$ such that:

$$\phi_1(i^*, j) \leq \frac{\phi}{h}. \tag{1}$$

The idea in the remaining proof is that z_2 hyperedges are definitely pushing flow outside of S, while z_1 hyperedges can be pushing flow inside and outside of S.

Consider any hyperedge counted in $z_2(i)$, these hyperedges have level difference between the node of the highest level and all other nodes of at least two, which means the residual capacity of the hyperedge in $z_2(i)$ is zero (Because the difference in level is at least two, this means the node of highest level in the hyperedge did not consider pushing flow to the hyperedge, this can be either because (1) It did not have excess of

flow, (2) a node in the hyperedge has reached its maximum capacity or (3) the hyperedge has reached its maximum capacity. Option (1) is not correct as the difference in level is at least two, which means the node with the highest label had excess and raised its level to push flow to another hyperedge. Additionally, option (2) is not correct as in this case, the node with maximum capacity has excess of flow and will end with level h making it the node with the highest level or it will raise its level and push flow to the hyperedge first and get space since it has the lowest level. Therefore, option (3) is the correct one and the hyperedge residual capacity is zero.) Since $i^* \geq \frac{h}{2} \geq \frac{1}{\phi}$, then $\min(\mathbf{l}(v), C)$ where v is the node pushing the flow across the hyperedge and as all nodes level is at least $\frac{h}{2}$, which is greater than $\frac{1}{\phi}$ and $C = \frac{1}{\phi}$, therefore, the flow of hyperedge f must be $\frac{1}{\phi}$. Hence pushing flow outside of S_i^* of $\frac{1}{\phi}$ per node. However, unlike the edge case, we cannot assume that all nodes of $z_2(i)$ is pushing flow from S_i^* to $\bar{S_i^*}$ as some of the nodes is actually inside S_i^* or inside $\bar{S_i^*}$. The $z_1(i)$ can push a maximum of $\frac{1}{\phi}$ in S (As an upper bound of the flow leaking outside S_i^*, we will assume that all edges of $z_1(i)$ is pushing flow into S_i^*) and $2\text{volM}(S_i^*)$ mass can start at S_i^*. Therefore, we have:

$$\text{Flow out of } S_i^* = \frac{\alpha z_2(i^*)}{(k-1)\phi}$$

$$\text{Flow out of } S_i^* \leq \frac{\sum_{j=i^*}^{h} z_1(i^*, j)}{\phi} + 2\text{volM}(S_i^*), \tag{2}$$

where α is the average number of nodes of $z_2(i^*)$ group that is on the other side $\bar{S_i^*}$. By assuming that S_i^* is the smaller side of the cut, we get:

$$\phi_M(S_i^*) = \frac{|cut_M(S_i^*, \bar{S_i^*})|}{\min(\text{vol}_M(S_i^*), \text{vol}_M(\bar{S_i^*}))}.$$

Multiply both the numerator and denominator by $k-1$, we get:

$$\begin{aligned}
\phi_M(S_i^*) &= \frac{\sum_{j=i^*}^{h} z_1(i^*, j) + z_2(i^*)}{\text{volM}(S_i^*)} \\
&\leq \frac{k-1}{\alpha} \frac{\sum_{j=i^*}^{h} z_1(i^*, j) + 2\phi\text{volM}(S_i^*)}{\text{volM}(S_i^*)} \\
&+ \frac{\sum_{j=i^*}^{h} z_1(i^*, j)}{\text{volM}(S_i^*)} \\
&\leq \frac{k-1}{\alpha}(\phi + 2\phi) + \phi \qquad \text{using inequality (2)} \\
&\leq \frac{3(k-1)}{\alpha}\phi + \phi \qquad \text{using inequality (1)} \\
&\leq (3k-2)\phi \qquad \alpha \in [1, k-1].
\end{aligned}$$

Hence, $\phi_M(S_i^*)$ is $\text{O}(k\phi)$. When $k=2$, the constant with ϕ is 4, which is exactly the constant in the original CRD proof. If S_i^* is not the smaller side of the cut, then similar to the CRD argument, we should run the contradiction argument from 1 to $\frac{h}{2}$ and note that at most $z_2/(k-1)$ by C are pushed into $\bar{S}_i$. This flow will either stay in $\bar{S}_i$ or go

back to S_i through z_1 hyperedges. Therefore:

$$\frac{z_2(i)}{(k-1)\phi} \leq \text{Flow in } \bar{S}_i^* \leq \sum_{u \in \bar{S}_i} m_M(u) + \frac{\sum_{j=i^*}^{h} z_1(i,j)}{\phi}$$
$$\leq \text{volM}(\bar{S}_i) + \frac{\sum_{j=i^*}^{h} z_1(i,j)}{\phi}.$$

Recall that $\bar{S}_i^*$ is the smaller side of the cut, we get:

$$\begin{aligned}\phi_M(S_i^*) &= \frac{\sum_{j=i^*}^{h} z_1(i^*,j) + z_2(i^*)}{\text{volM}(\bar{S}_i^*)} \\ &\leq \frac{\sum_{j=i^*}^{h} z_1(i^*,j)}{\text{volM}(\bar{S}_i^*)} \\ &+ \frac{(k-1)(\sum_{j=i^*}^{h} z_1(i^*,j) + \phi\text{volM}(S_i^*))}{\text{volM}(\bar{S}_i^*)} \\ &\leq (2k-1)\phi.\end{aligned}$$

Therefore, $\phi_M(S_i^*) = O(k\phi)$, which completes the proof. □

Lemma 6.2 (**Lemma 1 [30] Generalization**). *Let M_j be the total mass in B in the jth step of HG-CRD-inner and L_j be the total mass escaping B to $\bar{B}$, then we have: If $M_j \geq \frac{volM(B)}{2}$, then $L_j \leq O(\frac{1}{\sigma_1})M_j$ and if $M_j \leq \frac{volM(B)}{2}$, then $L_j \leq O(\frac{k}{\sigma_2 log\ vol_M(B)})M_j$.*

Proof. For case 1 of the proof, when $M_j \geq \frac{\text{vol}_M(B)}{2}$, we get:

$$\begin{aligned}L_j &\leq (k-1)|\text{cut}_M(B,\bar{B})|C \\ &\leq (k-1)\text{vol}_M(B)\phi_M(B)\frac{1}{\phi} && \text{using vol}_M(B) \leq \frac{\text{vol}_M(G)}{2} \\ &\leq 2M_j\phi_M(B)\frac{k}{\phi_M^{(S)}(B)} && \text{using } M_j \geq \frac{\text{volM}(B)}{2} \\ &\leq 2M_j\frac{1}{\sigma_1} = O(\frac{1}{\sigma_1})M_j && \text{Assumption 1.}\end{aligned}$$

For case 2, we have $M_j \leq \frac{\text{volM}(B)}{2}$. Let us define $B_i = \{v \in B | l(v) = i\}$ and $S_i = \{v \in B | l(v) \geq i\}$. As $M_j \leq \frac{\text{volM}(B)}{2}$, we have $\text{volM}(S_h) \leq ...\text{volM}(S_1) \leq M_j \leq \frac{\text{volM}(B)}{2}$. As nodes in S_i for $i = 1$ to h are either in case 0 or 1 of the levels and therefore they have $m_M(v) \geq d_M(v)$. Therefore, we can use

assumption 2 and get:

$$
\begin{aligned}
L_j &\leq (k-1)\sum_{i=1}^{h} |M(B_i,\bar{B})| \min(i, \frac{1}{\phi}) \\
&\leq (k-1)\sum_{i=1}^{h} \frac{|M(B_i, B\setminus B_i)|\frac{1}{\phi}}{\sigma_2 \ \log \ \mathrm{volM}(B)\frac{1}{\phi_M^{(S)}(B)}} && \text{Assumption 2} \\
&\leq \frac{(k-1)}{\phi}\sum_{i=1}^{h} \frac{\phi_M^{(B_i)}(B)\mathrm{vol}_M(B_i)}{\sigma_2 \ \log \ \mathrm{volM}(B)\frac{1}{\phi_M^{(S)}(B)}} && \text{using } \mathrm{vol}_M(B_i) \leq \frac{\mathrm{vol}_M(G)}{2} \\
&\leq \frac{1}{\phi}\sum_{i=1}^{h} \frac{\phi_M^{(B_i)}(B)\mathrm{volM}(B_i)}{\sigma_2 \ \log \ \mathrm{volM}(B)\frac{1}{\phi_M^{(S)}(B)}} && \text{using } (k-1)\sum_{i=1}^{h}\mathrm{vol}_M(B_i) \leq \sum_{i=1}^{h}\mathrm{m}_M(B_i) \leq M_j \\
&\leq M_j \frac{1}{\sigma_2 \ \log \ \mathrm{volM}(B)\frac{1}{\phi_M^{(S)}(B)}\phi} \\
&\leq O(\frac{k}{\sigma_2 \ \log \ \mathrm{volM}(B)})M_j,
\end{aligned}
$$

which completes the proof of the lemma. □

Theorem 4 (**Theorem 3 [30] Generalization**). *If we run HG-CRD with* $\phi \geq \frac{\Omega(\phi_M(B))}{k}$*, then we get:*

- $vol_M(A \setminus B) \leq O(\frac{k}{\sigma}).vol_M(B)$,
- $vol_M(B \setminus A) \leq O(\frac{k}{\sigma})vol_M(B)$,
- $\phi_M(A) \leq O(k\phi)$,

where $A = \{v \in V | d_M(v) \leq m_M(v)\}$ *and* $\sigma = min(\sigma_1, \sigma_2)$.

Proof. As $\phi \geq \frac{\Omega(\phi_M(B))}{k}$ and from theorem 1, we have $\phi_M(A) \leq O(k\phi)$, therefore we will get $\phi_M(A) \leq \phi_M(B)$ and therefore the diffusion will not stuck on any bottleneck subset inside B and will be able to spread the mass all over B.

Before all nodes in B are saturated, the leakage according to lemma 2 is $O(\frac{k}{\sigma \log \ \mathrm{volM}(B)})$ and we need log volM(B) iterations to saturate all nodes in B and therefore the leakage to $\bar{B}$ in all iterations is $O(\frac{k}{\sigma})$ fraction of the total mass in the graph before and after saturating the nodes in B.

After saturating nodes in B, we will run constant number of iterations before terminating and therefore at termination the total mass will be after removing excess of flow is $\theta(\mathrm{volM}(B))$ because at $t = \log \ \mathrm{volM}(B)$, the termination condition will be $\tau 2 d_M(v_s)\mathrm{volM}(B)$ and after log volM(B) all the nodes in B are saturated and the leakage is $O(\frac{\mathrm{volM}(B)}{\sigma})$ therefore, the total mass will be $\leq 2\frac{1+\sigma}{\sigma}\mathrm{volM}(B)$ and therefore the total mass is less than the termination condition by choosing appropriate τ. Hence, the upper bound for $\mathrm{vol}_M(A \setminus B)$ is: $\mathrm{vol}_M(A \setminus B) \leq (k-1)\mathrm{m}_M(A \cap \bar{B}) \leq O(\frac{k}{\sigma})\mathrm{vol}_M(B)$, which concludes the proof of case 1. For case 2, we get:
$\mathrm{vol}_M(B \setminus A) = \mathrm{vol}_M(B \cap \bar{A}) \leq O(\frac{k}{\sigma})\mathrm{vol}_M(B)$, as the total leakage is $O(\frac{\mathrm{volM}(B)}{\sigma})$. □
