# Supplementary material for: Local hypergraph clustering using capacity releasing diffusion
Source: PLoS One. 2020 Dec 23;15(12):e0243485. doi: 10.1371/journal.pone.0243485 (PMC7757905; doi:10.1371/journal.pone.0243485)
Supplement: Attachment — Submitted filename: refereePlos.pdf [file pone.0243485.s004.pdf]

Referee Report

Local hypergraph clustering using capacity releasing diffusion
by R. Ibrahim and D.F. Gleich
submitted to PlosOne
Manuscript PONE-D-20-27457

October 7, 2020

In this manuscript the authors place their research within the context of existing literature and extend the recent technique called CDR (capacity releasing diffusion, a method to find local clusters presented in 2017) to a new method called hypergraph CRD (HG-CRD). This new method outperforms the capabilities of the forementioned CDR in the sense that it provides a more robust computation of the conductance associated to the used motifs. The new method is important for two reasons. On the one hand, this new framework makes use of an hypergraph (or multigraph) defined on the same nodes that the original graph in which is wanted to perform the clustering, jointly with a set of hyperedges (sets of nodes connected by a pattern or motif). On the other hand, HG-CRD does not need the construction of the motif-weighted adjacency matrix (and this is a difference with some methods for local clustering based on Personalized PageRank). The authors also extend some known theoretical results to the new framework. One of the great capabilities of the presented method is the ability to use, in principle, any kind of motif, including directed subgraphs. The algorithm is not subjected to the Cheeger inequality when there is a proper cluster close to the seed nodes where the algorithm starts. The results are obtained by using synthetic and real graphs and show that HG-CRD properly finds motif clusters and it may be a useful tool for community detection in a wide range of applications.

The manuscript gives access to the datasets and codes. It is well organized and written but some minor typos and certain arrangements of equations should be corrected.

- Page 6, line 21. "Based on [13] proof ...". This sentence should be rewritten.
- Page 7. Line 10. The equation for $\mathbf{d}_M(v)$ does not appear to be elegant. Instead of this equation it could be defined something like $\eta(v)$ to be the number of hyperedges containing v and therefore $\mathbf{d}_M(v) = (k-1)\eta(v)$. In this form the manuscript can avoid lines 11 and 12.
- Page 7. Line 19. $e = (v, u_1, \dots u_{k-1})$ should be corrected to $e = (v, u_1, \dots, u_{k-1})$.

- Page 7. Line 24. $e = (v, u_1, \dots u_{k-1})$ should be corrected to $e = (v, u_1, \dots, u_{k-1})$.
- Page 8, line -14. "(Recall that ...". Should be "(recall that ..."
- Page 8, line -4. "(Even those ...". Parenthesis should be omitted
- Page 11, line 7. "edges and not true ...". This sentence should be rewritten.
- Page 17, line 16. There are two inequalities in the same line. These inequations should be rearranged.
- Page 19, lines 4 to 7. Proof of Lemma 6.2. These inequations should be properly rearranged and commented.
- Page 19, lines 13 to 20. Proof of Lemma 6.2. These inequations should be properly rearranged and commented.
- Page 19, line -2. " $vol_M(B \setminus A). \leq$" should be "$vol_M(B \setminus A) \leq$"
- Page 20, line -18. Correct reference number 2 (delete "null")
- Page 23. Table 1 should be rearranged.

To summarize, the proposed method is technically sound and represents a contribution in its area. All in all, I consider that this manuscript is worth publishing once the typos are corrected.
