# Supplementary material for: Local hypergraph clustering using capacity releasing diffusion
Source: PLoS One. 2020 Dec 23;15(12):e0243485. doi: 10.1371/journal.pone.0243485 (PMC7757905; doi:10.1371/journal.pone.0243485)
Supplement: Attachment — Submitted filename: Review.pdf [file pone.0243485.s005.pdf]

Review on Paper: Local hypergraph clustering using capacity releasing diffusion

October 18, 2020

The authors propose a generalization of the capacity releasing method to hypergraphs. I like that paper, my major concern is with the experiments. See my comments below. The proofs are fine, nothing surprising of course, but good to have them.

1 Comments for the main paper

- Page 1, second paragraph of the introduction. The sentence "These difference", should be "These differences".
- Page 7, the first bullet point before Subsection 4.3. The role of α here is confusing, what do you mean "for at at least α"?
- Figure 2. It's not clear from the figure which are the hyper-edges.
- Lemma 6.1 in the Supplementary material. There are both $volM$ and volM in this lemma and the proof.
- Theorem 3 in the Supplementary material. There are two cases in the description of the theorem and three cases in the proof, the same name is used twice for cases 1 and 2.

2 Comments for the experiments

- It's not clear from the text or the tables if you illustrate averaged results over all 100 communities for each dataset.
- How did you tune APPR and MAPPR?
- Choosing out of all node-trials the one that has the best F1-score does not show robustness. You need to show average results over the nodes as in the original capacity releasing diffusion paper.
- The communities that were used from DBLP, Amazon and YouTube seem to be extremely noisy to the point that I cannot even rely on the presented numbers. Especially for DBLP and YouTube the F1 score is 0.359

and 0.175, respectively. The methods barely detect anything. It might be because the authors simply averaged the performance over all 100 communities (to be verified above). The authors need to find some representative communities from these datasets and demonstrate performance on the representative communities. The same holds for Email-EU, average performance seems to be around 0.5-0.6 for all motifs. The same holds for the experiments in Table 6. These cannot be reliable numbers, best performance is less than 0.5.
