# Supplementary material for: Local hypergraph clustering using capacity releasing diffusion
Source: PLoS One. 2020 Dec 23;15(12):e0243485. doi: 10.1371/journal.pone.0243485 (PMC7757905; doi:10.1371/journal.pone.0243485)
Supplement: Attachment — Submitted filename: PLOS_one_reply.pdf [file pone.0243485.s006.pdf]

Local hypergraph clustering using capacity releasing diffusion

Rania Ibrahim and David F. Gleich

Computer Science Department, Purdue University, West Lafayette, Indiana, USA

* ibrahimr@purdue.edu

We would like to thank the reviewers for the comments and time to improve our paper. Please find our reply for each point raised by the reviewers. Also, see the attached *diff* that shows how these changes have impacted the manuscript.

The largest change is the addition of a new *robustness* section in response to Reviewer Two's comments on the performance of the method. This shows numerous scenarios where our method does quite well.

1 Reviewer One

- **Page 6, line 21. "Based on [13] proof ...". This sentence should be rewritten.**

  Done.

- **Page 7. Line 10. The equation for dM(v) does not appear to be elegant. Instead of this equation it could be defined something like (v) to be the number of hyperedges containing v and therefore dM(v) = (k 1)(v). In this form the manuscript can avoid lines 11 and 12.**

  Done.

- **Page 7. Line 19. e = (v, u1, . . . uk1) should be corrected to e = (v, u1, . . . , uk1).**

  Done.

- **Page 7. Line 24. e = (v, u1, . . . uk1) should be corrected to e = (v, u1, . . . , uk1).**

  Done.

- **Page 8, line -14. "(Recall that ...". Should be "(recall that ..."**

  Done.

- **Page 8, line -4. "(Even those ...". Parenthesis should be omitted**

  Done.

- **Page 11, line 7. "edges and not true ...". This sentence should be rewritten.**

  We have rewritten the sentence.

- **Page 17, line 16. There are two inequalities in the same line. These inequations should be rearranged.**

  Done.

- **Page 19, lines 4 to 7. Proof of Lemma 6.2. These inequations should be properly rearranged and commented.**

  We have revisited the inequalities.

- **Page 19, lines 13 to 20. Proof of Lemma 6.2. These inequations should be properly rearranged and commented.**

  We have revisited the inequalities.

- **Page 19, line -2. " volM(B \A). " should be "volM(B\A) "**

  Done.

- **Page 20, line -18. Correct reference number 2 (delete "null")**

  Done.

- **Page 23. Table 1 should be rearranged.**

  We have separated the pseudocode and the table.

2 Reviewer Two

2.1 Comments for the main paper

- **Page 1, second paragraph of the introduction. The sentence "These difference", should be "These differences".**

  Done.

- **Page 7, the first bullet point before Subsection 4.3. The role of α here is confusing, what do you mean "for at at least "?**

  We have modified the sentence to illustrate examples of $\alpha = 1$ and $\alpha = 2$.

- **Figure 2. It's not clear from the figure which are the hyper-edges.**

  In this figure, we consider triangles as hyperedges; we added a clarification in the caption as well as other captions.

- **Lemma 6.1 in the Supplementary material. There are both volM and volM in this lemma and the proof.**

  These are typeset to match the surrounding text and the lemma/theorem statements are italic in the PLOS template.

- **Theorem 3 in the Supplementary material. There are two cases in the description of the theorem and three cases in the proof, the same name is used twice for cases 1 and 2.**

  The first two cases are for termination states for HG-CRD, while the cases in the proof are for levels. We have modified the proof to use the word "category" to avoid confusion.

2.2 Comments for the experiments

- **It's not clear from the text or the tables if you illustrate averaged results over all 100 communities for each dataset.**

  Yes, you are right we average the results over all 100 communities for each dataset. We have modified the text to mention this.

- **How did you tune APPR and MAPPR?**

  We have used the numbers reported in "Local Higher-Order Graph Clustering" KDD 2017 paper. We have modified the text to mention this as well.

- **Choosing out of all node-trials the one that has the best F1-score does not show robustness. You need to show average results over the nodes as in the original capacity releasing diffusion paper.**

  We follow the the evaluation setup used in "Local Higher-Order Graph Clustering" KDD 2017 paper. They start from each node and then report the result from the node that has the best F1. However, we understand your concern and we have added new experiments called "Robustness Experiments", where we report the average results over the nodes in the cluster along with the distribution of the results.

- **The communities that were used from DBLP, Amazon and YouTube seem to be extremely noisy to the point that I cannot even rely on the pre sented numbers. Especially for DBLP and YouTube the F1 score is 0.359 and 0.175, respectively. The methods barely detect anything. It might be because the authors simply averaged the performance over all 100 com unities (to be verified above). The authors need to find some representative communities from these datasets and demonstrate performance on the representative communities. The same holds for Email-EU, average performance seems to be around 0.5-0.6 for all motifs. The same holds for the experiments in Table 6. These cannot be reliable numbers, best performance is less than 0.5.**

  The same number ranges were reported by MAPPR in "Local Higher-Order Graph Clustering" KDD 2017 paper. These datasets contain hard to recover communities and therefore they serve as a useful – albeit noisy – reference point.

  However, we agree with your concern and we have added new experiments called "Robustness Experiments", where we have filtered the communities to only include clusters with conductance less than 0.5 and volume at least 1000. We also report the average results over the nodes in the cluster along with the distribution of the results. These are designed to be easier to find and highlight a case where our methods work better and a case where our methods “tie” (because everyone is essentially finding the perfect cluster).
